# The Microbiome of Peri-Implantitis: A Systematic Review of Next-Generation Sequencing Studies

**DOI:** 10.3390/antibiotics12111610

**Published:** 2023-11-09

**Authors:** Koay Chun Giok, Rohit Kunnath Menon

**Affiliations:** 1 School of Dentistry, International Medical University, Kuala Lumpur 57000, Malaysia; koay.chungiok@student.imu.edu.my; 2 College of Dentistry, Ajman University, Ajman 346, United Arab Emirates

**Keywords:** peri-implantitis, microbiome, sequencing, dental implant, complications

## Abstract

(1) Introduction: Current evidence shows that mechanical debridement augmented with systemic and topical antibiotics may be beneficial for the treatment of peri-implantitis. The microbial profile of peri-implantitis plays a key role in identifying the most suitable antibiotics to be used for the treatment and prevention of peri-implantitis. This systematic review aimed to summarize and critically analyze the methodology and findings of studies which have utilized sequencing techniques to elucidate the microbial profiles of peri-implantitis. (2) Results: *Fusobacterium, Treponema, and Porphyromonas* sp. are associated with peri-implantitis. *Veillonella* sp. are associated with healthy implant sites and exhibit a reduced prevalence in deeper pockets and with greater severity of disease progression. *Streptococcus* sp. have been identified both in diseased and healthy sites. *Neisseria* sp. have been associated with healthy implants and negatively correlate with the probing depth. Methanogens and AAGPRs were also detected in peri-implantitis sites. (3) Methods: The study was registered with the International Prospective Register of Systematic Reviews (PROSPERO) (CRD42023459266). The PRISMA criteria were used to select articles retrieved from a systematic search of the Scopus, Cochrane, and Medline databases until 1 August 2023. Title and abstract screening was followed by a full-text review of the included articles. Thirty-two articles were included in the final qualitative analysis. (4) Conclusions: A distinct microbial profile could not be identified from studies employing sequencing techniques to identify the microbiome. Further studies are needed with more standardization to allow a comparison of findings. A universal clinical parameter for the diagnosis of peri-implantitis should be implemented in all future studies to minimize confounding factors. The subject pool should also be more diverse and larger to compensate for individual differences, and perhaps a distinct microbial profile can be seen with a larger sample size.

## 1. Introduction

Dental implants exhibit high success rates of up to 97% and above [1]. However, contributory factors related to occlusal overloading and peri-implant tissue infection may lead to implant failure [2]. Peri-implantitis is defined as an infection of the peri-implant tissues accompanied by suppuration and clinically significant progressive crestal bone loss after the adaptive phase, leading to decreased osseointegration and pocket formation [3,4]. Peri-implantitis has a reported prevalence ranging from 6.6% to 51% [5,6,7,8,9]. Various risk factors are associated with an increased risk of peri-implantitis. Prosthetic factors, including convex emergence profiles, submucosal crown margins, and excess cement in cemented implant prostheses, increase the risk of peri-implantitis [2,3]. Systemic conditions such as diabetes mellitus and osteoporosis also increase the risk of peri-implantitis [10]. Furthermore, smoking has been found to directly affect the bone surrounding the implant, thereby increasing the risk of peri-implantitis as well [11]. Biofilm removal and control with instruments such as Gracey curettes, ultrasonic scalers, and air powder abrasive devices have been employed with questionable success in the treatment of peri-implantitis since mechanical debridement also comes with its challenges, especially at the apically facing thread surfaces, as demonstrated by Steiger-Ronay et al. [12]. Antimicrobials are also ineffective if mechanical debridement is inadequately performed, as mentioned previously [13,14]. However, liquid desiccants have been reported to reduce the anaerobic bacteria load in diseased implants [15]. To date, the treatment of peri-implantitis is similar to that of periodontitis [16]. The prognosis of this condition is uncertain, and hence, determining the fundamental cause is important for preventive strategies and also targeted approaches [17].

The exact mechanism of microbial interaction in peri-implantitis is not clearly known [3]. Initial studies reported that Staphylococcus aureus plays a role in the progression of the disease [18,19]. However, the consensus on the predominance of S. aureus in peri-implantitis sites was contradicted by Belibasakis et al., as their study concluded the predominance of Treponema spp. and Synergistetes cluster A in peri-implantitis sites [19,20].

Koyanagi et al. reported a more diverse microbial profile compared to that of periodontitis [21], while other studies indicated similarity [22,23]. A microbial profile consisting of aggressive and resistant microorganisms distinct from periodontitis has also been reported previously [24]. Periodontally involved teeth act as reservoir for periopathogens which translocate to the implant sites, making chronic periodontitis an important risk factor for peri-implantitis [21,23,25,26].

Culture-dependent studies evaluating the microbiome of peri-implantitis have limited insights into the bacterial community [27,28], and more recent next-generation sequencing techniques may give us an insight into a more targeted approach to peri-implantitis treatment which, in turn, can improve the prognosis of this condition [29]. The use of next-generation sequencing allows the identification of non-culturable species as compared to conventional methods [29]. The detection of bacterial and fungal infections has been shown to be consistently accurate as compared to conventional methods [30]. In addition, next-generation sequencing has been shown to be cost-effective for identifying the disease with a given high pretest probability, as compared to culture methods [31].

This systematic review aims to summarize and critically analyze the methodology and findings of studies that have utilized next-generation sequencing techniques to elucidate the microbial profiles of peri-implantitis.

## 2. Results

**Figure 1:**
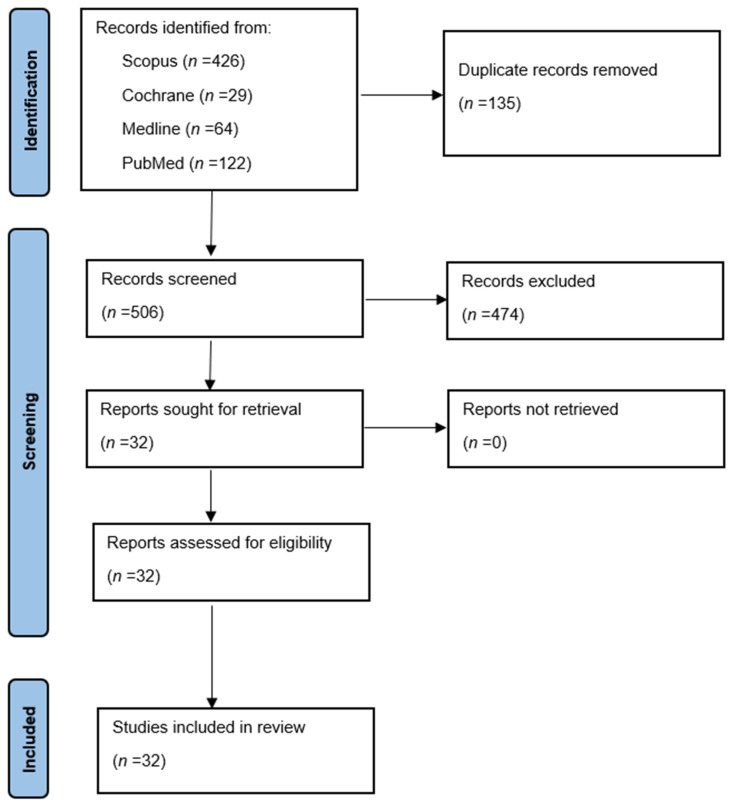
PRISMA flowchart.

From the initial search, 506 articles were identified after the elimination of duplicates. After performing the preliminary review of the title and abstracts, 32 articles were included for full-text screening. Based on the selection criteria, 32 studies were chosen to be included in the qualitative analysis (Figure 1). The Risk Of Bias In Non-randomized Studies–of Exposures (ROBINS-E) assessment of 32 articles is shown in Table 1. The Grading of Recommendations, Assessment, Development, and Evaluation (GRADE) approach was used (Table 2) and revealed a low certainty of evidence for the outcomes of diversity and richness as well as the abundance of taxa.

### 2.1. Methodology of Studies

The methodological characteristics of the studies published between 2009 and 2021 are depicted in Table 3. The total sample size of the selected studies ranged from two to one hundred and six. Fifteen studies compared the association of the periodontitis microbiome with the peri-implantitis site microbiome [21,22,25,32,35,39,41,42,45,47,48,49,51,54,57]. Twelve studies compared the microbiomes of healthy implant (HI) sites to those of peri-implantitis (PI) sites [20,33,34,40,43,44,46,51,53,55,56,58], where the healthy implant site was the control. Peri-implant mucositis (PM) was also compared to peri-implantitis in seven studies [36,37,40,47,50,52]. Smoking was investigated as a factor in microbial dysbiosis in two studies [49,50]. Furthermore, Kroger et al. [43] investigated the association between the microbial diversity and the pocket depths of implants, while Korsh et al. [38] investigated the microbiota associated with early versus late implant loss.

Oral samples collected for microbiome isolation in the 32 included studies were composed mostly of subgingival plaque samples [20,21,22,23,25,32,33,35,36,37,38,39,40,41,42,43,44,45,46,47,48,49,50,51,52,53,54,55,56,57,58]. Two studies utilized supragingival plaque samples [32,53]. Sterile paper points were used to collect the subgingival plaque samples [21,22,23,25,33,35,36,37,38,39,42,43,44,46,48,49,50,51,53,54,55,57]. Eight studies utilized sterile Gracey curettes [20,32,40,41,45,47,56,58], while one study used a periodontal probe [52]. Further details on the collection method are provided in Table 3.

The DNA extraction technique, sequencing technique, targeted region, and the reference database for each study are summarized in Table 4. The microbiome profile is depicted in relation to the diversity, richness, and taxa abundance in Table 5.

Among the 32 studies reviewed, seven studies found an increase in the microbial diversity of peri-implantitis sites as compared with healthy implant sites [20,23,33,38,43,44,52]. Five studies did not report the diversity and richness of the samples collected [41,46,51,55,56,58]. Five studies reported an increase in the microbial diversity in peri-implantitis sites as compared with periodontitis sites [21,32,35,39,57]. Five studies reported a reduced microbial diversity in peri-implantitis sites compared with healthy implants in subgingival plaque [22,34,44,45,52]. Additionally, four studies reported no significant difference in diversity between healthy implants and peri-implantitis samples [23,33,37,50].

### 2.2. Microbial Profile

Koyanagi et al. revealed that implants with peri-implantitis had a higher abundance of *Eubacterium* spp. when compared to healthy implants, and this finding is also supported by Zheng et al. and Kroger et al. [21,43,52]; da Silva et al. found that healthy implants demonstrated lower proportions of *Eubacterium* compared to peri-implantitis sites, while Koyanagi et al. and Zheng et al. concluded that peri-implantitis sites had significantly higher proportions of *Eubacterium* [21,52,56]. Sanz-Martin et al. reported higher levels of *Eubacterium* in a healthy implant, when a diseased implant was also present in the same oral cavity [20]. Two studies found high levels of *Bacteroidetes* and *Firmucutes* in PI sites as compared to HI sites [20,46]. Three authors found higher levels of *Bacteroides* in diseased implants [32,33,34]. Yu et al. demonstrated that *F. fastidiosum SH03* and the *Fretibacterium* oral taxon *SH01* were linked with plaque at healthy subgingival sites [48]. This study concluded that there were no clear differences or similarities between *Synergistetes* communities found in diseased versus healthy sites or between periodontal/subgingival niches and peri-implant/submucosal niches [48]. Another study by Yu et al. also showed that the prevalent and abundant bacteria were *Streptococcus infantis/mitis/oralis (HMT-070/HMT-071/HMT-638/HMT-677)* and *Fusobacterium* sp. *HMT-203/HMT-698* in healthy implants and diseased implants [42]. Another 18 phyla were found in low abundance, particularly the *Aquificae*, *Chlamydiae*, *Gemmatimonadetes*, *Nitrospirae*, *TM6*, *Verrucomicrobia*, and *WPS2* phyla, which were present in <0.01% of the total reads for each of the four clinical site categories, with some being undetectable in one or more niches [42]. Healthy implants demonstrated higher proportions of *Actinomyces, Atopobium, Gemella, Kingella* and *Rothia* and lower levels of *Campylobacter*, *Desulfobulbus, Dialister, Eubacterium*, *Filifactor, Mitsukella, Porphyromonas,* and *Pseudoramibacter* in one study [56]. One study that underwent a pathogen-specific analysis for *Archaea* found that PI sites had a higher frequency of sites that were positive for *Archaea* [58]. *Filifactor* was found to be abundant in peri-implantitis sites when compared with healthy implant sites, as shown by several studies [20,35,36,40,47,55,56]. Three studies demonstrated that *Parvimonas* was the most abundant at peri-implantitis sites [21,55,57].

#### 2.2.1. Phyla

The range of phyla was reported to be varied among the 25 studies. Koyanagi T et al. reported that *Firmicutes* (45.6%) is the most abundant phylum found in the subgingival plaque in peri-implantitis samples, followed by *Bacteroidetes*, *Proteobacteria, Fusobacteria*, *Actinobacteria*, TM7, *Synergistetes*, *Spirochaetes*, *Tenericutes*, *Chloroflexi*, and *Deferribacteres* [21]. Three studies were in concordance in concluding that *Bacteroidetes* is one of the genera that is found in great abundance in peri-implantitis samples [20,21,46]. The abundance of *Synergistetes* was reported to be higher in diseased samples in four studies in comparison to in healthy samples [20,21,23,33]. *Spirochaetes* was identified in diseased samples in three studies [20,21,46], with one study reporting that *Spirochaetes* increased significantly as peri-implantitis became more severe [20].

#### 2.2.2. Genus

Numerous changes were reported at the genus level (Table 5), with many of them focusing on several genera which are the most abundant in the peri-implant sites. One study reported that there was a preponderance of *Veillonella* in diseased peri-implant mucosal tissues [45]. However, there are also studies that have suggested that *Veillonella* is significantly reduced in samples with an increasing peri-implantitis severity [20,53]. *Veillonella* was also associated with healthy implant sites in other studies [20,47,55,56]. Several authors have found that *Prevotella* spp. are significantly more abundant at peri-implantitis sites [23,34,36,39,53,54]. Kumar et al. and Daubert et al. found that healthy implants showed higher levels of these two microorganism species [22,45], which was also supported by Apatzidou et al., who showed their greater abundance in diseased samples [23]. Other than *Veillonella* and *Prevotella*, most studies also pointed out that *Porphyromonas* was commonly associated with diseased implants [20,23,51,53,56]. Several studies pointed out that *Fusobacterium* was present in high levels in peri-implantitis samples [21,37,41,46,55,56,57]. Five studies reported that *Streptococcus* was more abundant in healthy plaque samples as compared to its abundance in diseased samples [20,22,23,44,45]. Yu et al. also found that *Streptococcus* was found in both healthy implants and peri-implantitis sites [42]. On the contrary, Kumar et al. concluded that peri-implantitis samples demonstrated a higher level of *Streptococcus* [22]. A study reported that *Propionibacterium*, *Paludibacter*, *Staphylococcus*, *Filifactor*, *Mogibacterium*, *Bradyrhizobium*, and *Acinetobacter* are unique to peri-implant sites [47]. In addition, *Actinomyces* spp. has been reported to be prevalent in peri-implantitis sites [22,52,53]. However, da Silva et al. reported higher levels of *Actinomyces* spp. in healthy implants [56].

#### 2.2.3. Microbiome Complex

Apart from the genera and phyla levels, Al-Ahmad et al. and Kim et al. reported that *Porphyromonas gingivalis* and *Tannerella forsythia* of the red complex are highly associated with peri-implantitis [32,46]. A study reported positive correlations with certain red and orange complex bacteria but a negatively correlation with blue complex bacteria in peri-implantitis samples [20]. Furthermore, another study reported that *Bacteroidetes, Chloroflexi, Spirochaetes, Synergistetes,* and *TM7* positively corresponded with the pocket depths [23].

#### 2.2.4. Peri-Implantitis with Periodontitis

*Granulicatella adiacens* (phylum *Bacillota*) was identified in two-thirds of peri-implantitis sites; these two species were also detected at periodontitis sites but not in healthy implants [57]. Shiba et al. found that the microbial composition at the genus level was diverse among the samples for each disease and between both samples from each individual, although the predominant species were similar [49]. Two studies showed that the periodontitis microbial community is more diverse than peri-implantitis sites [25,47]. Interestingly, three studies found the opposite, whereby periodontitis samples yielded lower diversities than peri-implantitis samples [21,22,57]. Aleksandrowicz et al. demonstrated that *Archaea* was found in diseased implants and teeth [41]. Furthermore, they were found in abundant levels at periodontitis sites when compared to peri-implantitis sites [41].

#### 2.2.5. Peri-Implantitis with Peri-Implant Mucositis

Shi et al. reported no differences in diversity between peri-mucositis sites as compared to peri-implantitis sites, but they found an increased microbial richness in peri-mucositis sites [36]. Sousa et al. reported a decreased abundance of *Bradyrhizobium* in peri-mucositis sites and peri-implantitis sites [47]. One study concluded that the microbial profile associated with peri-implantitis was also present with a moderate relative abundance at peri-mucositis sites. This study also found that the Shannon index of peri-mucositis was lower than that of peri-implantitis [52]. Tsigarida et al. reported subtle differences between the peri-mucositis and peri-implantitis microbiomes, and these subtle differences were between the transition from health to disease [50]. *Streptococci* and *Rothia* were associated with peri-mucositis, while *Fusobacterium* and *Treponema* were associated with peri-implantitis, as shown by Polymeri et al. [37]

### 2.3. Heterogeneity of Studies

**Figure 2:**
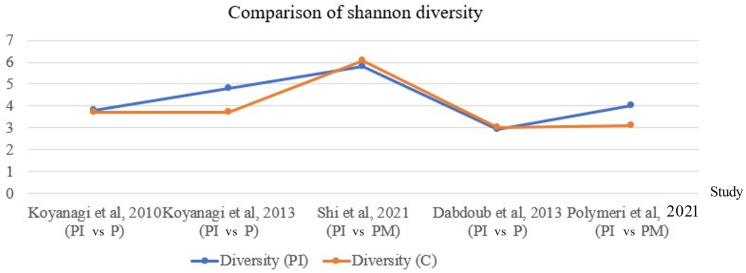
Different Shannon’s indexes reported by the studies reviewed. PI—peri-implantitis, PM—peri-implant mucositis, P—periodontitis, C—comparison group [21,25,36,37,57].

**Figure 3:**
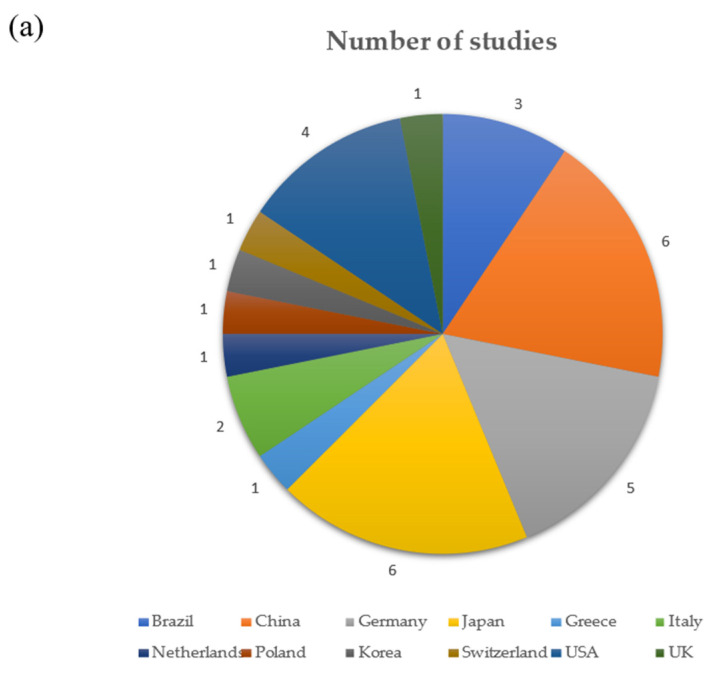

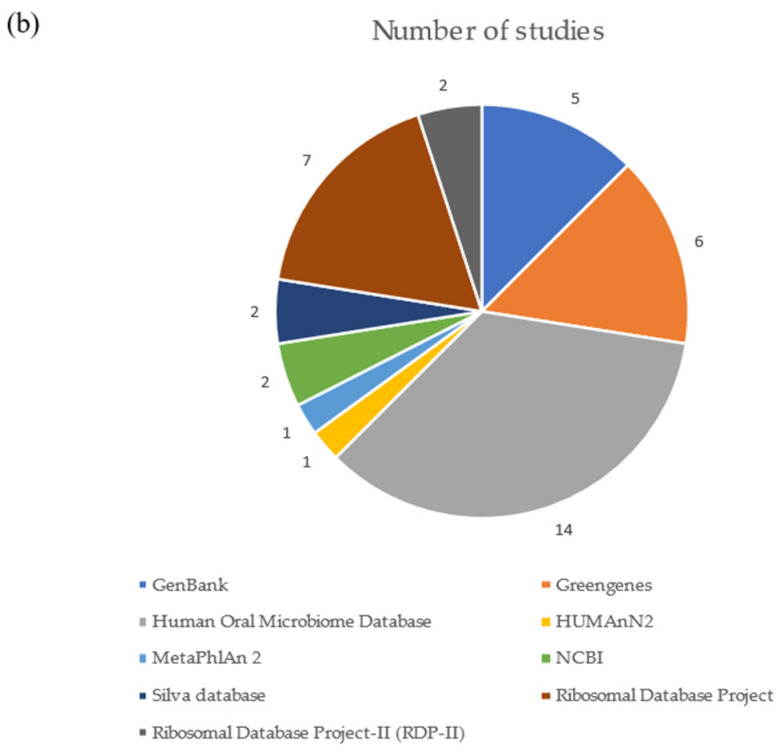

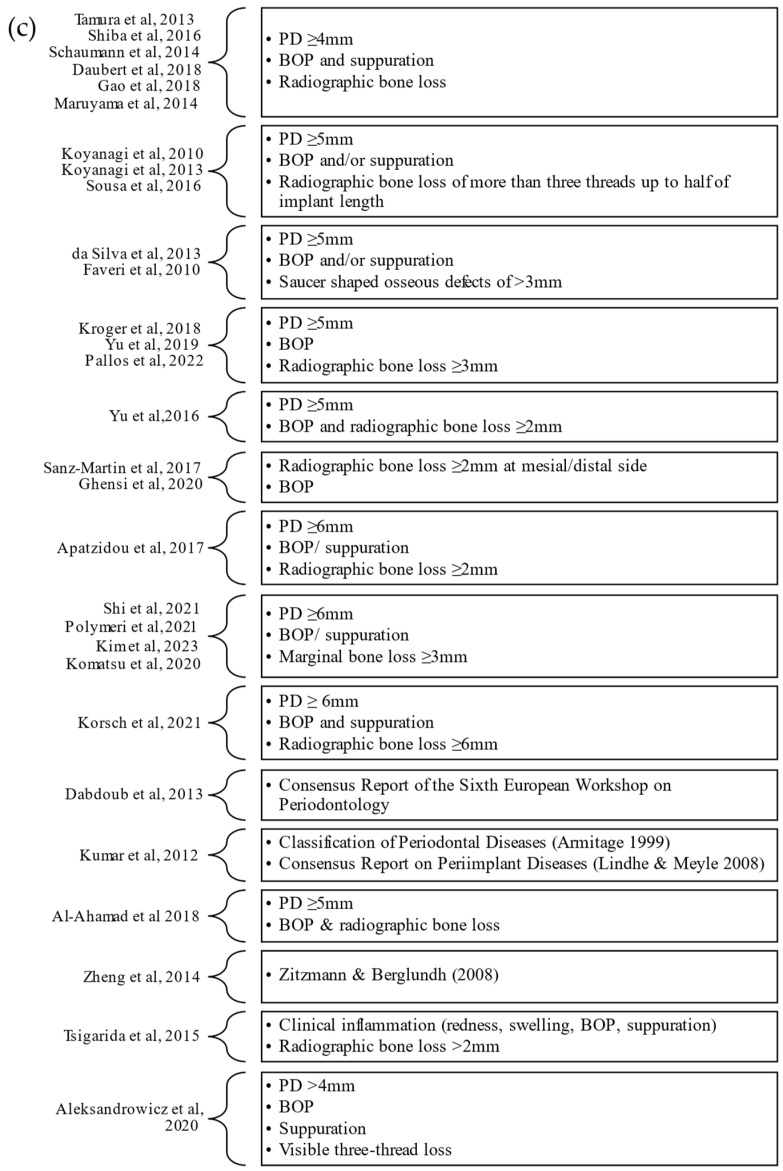
(**a**) Location; (**b**) database used; (**c**) case definition criteria of the studies reviewed. PD: probing depth; BOP: bleeding on probing [20,21,22,23,25,32,34,36,37,38,39,40,41,42,43,44,45,46,47,48,49,50,52,53,54,55,56,57,58].

Significant heterogeneity can be identified in the methodologies of the selected studies. The ROBINS-E tool was used to assess the quality of the 32 nonrandomized cohort observational studies. The ROBINS-E tool (Table 1) showed that nine studies had some concerns, while four studies were at a high risk of bias. Table 4 illustrates the heterogenicity of the gene sequencing techniques utilized. Figure 2 illustrates the diversity reported in terms of the Shannon’s indexes reported by five studies [21,25,36,37,57]. Figure 3 illustrates the heterogeneity regarding the location (Figure 3a), database used (Figure 3b), and case definition criteria (Figure 3c) of the studies reviewed.

## 3. Discussion

This systematic review comprehensively reviews the current available evidence on the microbiome of peri-implantitis. Variations in the study methods, sample collection, and study design were observed. However, the review focuses on studies employing the 16S r RNA gene sequencing technique to summarize meaningful observations from the available evidence.

Ten of the studies reviewed showed that the microbial diversity of peri-implantitis is distinct and usually higher than that at healthy implant sites [14,15,17,19,24,26,28,34,38,39]. The alpha diversity considers the richness (number of taxa) and evenness (relative abundance) of species within a sample/community; the beta-diversity quantifies the identities of taxa involved between samples/communities [49]. Changes in oxygen and nutrient concentrations associated with the deepening of a pocket around an implant may be responsible for the shift in the microbial diversity [32]. Figure 2 shows the Shannon’s indexes reported by five studies, as not all studies reported indices [21,25,36,37,57]. These variations in the diversity can be explained by the heterogenicity of various factors such as the location of the study (Figure 3a), the reference database (Figure 3b), and the case criteria definition (Figure 3c). A variation in the genomic database can introduce conflicting results, as one study showed that even the use of a single database within a study can implicate systematic errors during the mapping process which subsequently affects genomic analyses [59]. In addition to that, the sample collection method and the type of sample collected are other confounding factors that may produce conflicting findings.

The studies that included in the current review originate from different countries (Figure 3a), for example, Japan [21,49,55,57], China [36,42,48,52,60], United States of America [22,25,45,50], United Kingdom [47], Germany [38,43,46,53], and The Netherlands [37]. It is significant to note that certain sections of the globe are not represented here. This may also be due to the exclusion of articles written in other languages. Hence, the current data may be significantly influenced by the diet and genetic make-up of the individuals from the representative countries [61]. The characterization of oral dysbiosis in different ethnicities and races presents significant challenges due to variations across multiple studies [62,63,64]. This is due to the highly varied diet, nutrition and lifestyle practices present over several generations in different geographical locations [65,66].

The case definition for peri-implantitis varied across the studies reviewed (Figure 3c). For example, Koyanagi et al. used a criteria of a probing depth (PD) ≥5 mm with bleeding on probing (BOP) and/or suppuration and bone loss >3 threads up to half of the implant length, while Apatzidou et al. diagnosed subjects as having peri-implantitis when there was PD ≥ 6 mm, BOP and/or suppuration, and radiographic bone loss of ≥2 mm in at least one implant surface after one year of loading [21,23]. However, it is evident that the disease severity may vary, even with the employment of the above criteria, hence making it difficult to combine or compare the results of certain studies. Standardizing the methodological quality of microbiome studies has been previously suggested as a necessary step in this direction.

Even though few studies included criteria related to the systemic status of the patient, drugs taken, previous history of other oral diseases like periodontitis and the age of the patient into consideration, the varied criteria set across studies makes a meaningful comparison irrelevant. It would be greatly beneficial for future investigations into the microbiome of the oral cavity to follow a standardized protocol to facilitate comparability between studies [67].

The reviewed studies provide a deeper understanding of the microbial profile of peri-implantitis. However, the different DNA extraction kits used may have had an influence on the microbial data, for example, the Qiagen DNA MiniAmp kit, (QIAGEN, Venlo, The Netherlands) [22,25,38,42,48,50,53], GenElute Bacterial Genomic DNA kit, (Sigma-Aldrich, Munich, Germany) [43], Mora-extract kit, (AMR Inc., Tokyo, Japan) [21,57], Real-time PCR with TaqMan Probe, (Thermo Fisher Scientific, Waltham, MA, USA) [23], DNeasy Kit, (QIAGEN, Venlo, The Netherlands) [36,46], and the Masterpure purification kit, (Epicentre, Verona, Wisconsin, USA) [20,56].

Despite being considered an extension of peri-implantitis and the presence of common bacteria, peri-implant mucositis has been reported to have a distinct microbial profile in some studies [68,69]. However, a few studies were not able to provide a conclusive result on this aspect [36,37,47,50,52]. The diversity in peri-implant mucositis has been reported to be higher than at healthy implant sites [36] but lower than in peri-implantitis [52]. Moreover, the immune cell profiles of both entities seem to differ as well. Enhanced neutrophil and B-cell responses have previously been identified for peri-implantitis lesions when compared to peri-implant mucositis lesions under experimental conditions. The shift in the microbiome profile may also be explained by the increase in frequency and the number of bleeding sites subsequent to biofilm accumulation surrounding the implants [70].

The association of *Veillonella* sp. with healthy implant sites is well-correlated with its reduced prevalence in deeper pockets and severe disease progression [20,43,46,55]. *Streptococci* spp. have been identified in both diseased [21,22,53,56] and healthy sites [20,23,45]. *Neisseria* sp. have been associated with healthy implants and negatively correlates with the probing depth [20,40,43,44], suggesting that *Neisseria* sp. could have been replaced by other colonizers or may exert a protective effect. Species of the genus *Neisseria* are well-established primary colonizers of the dental plaque of natural teeth but are not well known for their presence in dental implants. On the contrary, three studies reported high levels of *Neisseria* sp. in peri-implantitis sites, which contradicts other studies [22,51,54]. Considering the common occurrence of these species in the oral cavity and the possibility of transfer from a diseased to a healthy site or vice versa leads to the lack of a clear understanding of its role in the initiation and the progression of the disease.

Numerous studies have identified *Fusobacterium* sp. as the dominant species in peri-implantitis [20,21,46]. Studies have also reported the presence of the genus *Treponema* at peri-implantitis sites of increasing severity [20,43]. However, Kumar et al. reported higher levels of the genera *Treponema* and *Prevotella* at healthy implant sites, which is the opposite to what other studies have found [22]. Peri-implantitis sites have also seen an abundance of species from the phylum *Synergistetes* [20,23,46]. *Porphyromonas* sp. have been reported at peri-implantitis sites by multiple studies [20,21,23].

A distinct microbial pattern could not be identified across all the 25 studies reviewed, possibly due to the abovementioned factors. Sahrmann et al. also found that there was an absence of a characteristic bacterial profile at peri-implantitis sites [71]. Both the current review and the review by Sahrmann et al. had a consensus that there was considerable heterogeneity in the studies reviewed [71]. The red complex is frequently identified at peri-implantitis sites, as are putative pathogens of the orange and yellow complex. Furthermore, it seems that the relative abundance of each complex changes with an increasing disease progression severity. The blue complex was also reported to be negatively correlated with peri-implantitis sites, suggesting its protective effect. The red complex was also more abundant at implant sites for subjects who smoked, which correlates well with our current understanding that smoking is a risk factor for peri-implantitis. The studies have findings that contradict one another, and this makes it difficult to obtain a characteristic microbial profile for peri-implantitis. However, it is evident that the microbiome of peri-implantitis is unique and distinct from that of periodontitis.

Carvalho et al. found that peri-implantitis lesions were associated with the presence of *S. epidermidis, P. gingivalis, T. forsythia, T. denticola, F. nucleatum*, and *P. intermedia* [72]. The review included culture-dependent studies in the analysis. On the contrary, the current systematic review only included studies that utilized next-generation sequencing due to its improved detection limit [30,73]. Additionally, Carvalho et al. reported that a definitive conclusion regarding the microbiome of peri-implantitis could not be reached due to the nature of the studies analyzed. Next-generation sequencing methods have shown that the microbiome of peri-implantitis is distinct from that of periodontitis. Non-culturable species such as *Fusobacterium* and the *Treponema* sp. *HMT-257* have been detected in peri-implantitis lesions [74,75]. The current systematic review demonstrates that, even with the inclusion of only next-generation sequencing studies, a distinct and unique microbial community pattern could not be identified.

The current review is limited by the studies’ number of participants, with the highest being 139 in a study by Aleksandrowicz et al. [41]. This suggests that the results may not be generalized to the clinical setting due to the small sample size. This review is also limited by the heterogeneity presented across all studies reviewed. Hence, a characteristic microbial profile cannot be determined for future targeted therapies.

## 4. Materials and Methods

A systematic review of observational and case-control studies (PROSPERO) (CRD42023459266) investigating the microbiome of peri-implantitis lesions was performed on the Cochrane, Medline, and Scopus databases from inception until 1 August 2023 and reported according to the Preferred Reporting Items for Systematic Reviews and Meta-Analysis (PRISMA) [76]. A focused question was formulated based on PECO (population, exposure, comparator, and outcome). The population included patients with at least one osseointegrated dental implant, the exposure was the diagnosis of peri-implantitis lesions, the comparator included healthy implants, periodontitis sites, as well as peri-implant mucositis sites, and the outcome measure was the bacterial composition obtained from samples taken from peri-implantitis sites, as assessed through next-generation sequencing. The question was as follows: Among patients with at least one osseointegrated dental implant, what would be the difference between peri-implantitis lesions, healthy implants, periodontitis, and peri-implant mucositis in terms of the bacterial composition obtained from samples as assessed via next-generation sequencing?

The search strategy involved a combination of the following key terms: peri-implantitis, inflammation, disease, infection, consequence, sequence analysis, RNA, 16S, metagenomics, metagenome, microbiota, and bacteria. The keywords were combined using the Boolean operators “AND” and “OR” in the strategic search. This systematic review followed the Preferred Reporting Items for Systematic Reviews and Meta-Analyses (PRISMA) criteria [77].

The titles and abstracts were independently screened by two reviewers (K.C.G., R.K.M.) for eligible studies, followed by full-text reading. Data were extracted independently and in duplicate by the two reviewers (K.C.G., R.K.M.) into a data extraction form created following the Cochrane Handbook of Systematic Reviews of Interventions guidelines [76]. Observational and case-control studies investigating the microbiome of peri-implant tissues through next-generation DNA sequencing methods were included. Culture-based studies, conference papers, review articles, studies regarding peri-implantitis associated with other systematic factors (diabetes mellitus, immune disorders, etc.), and articles that examined only specific microorganisms were excluded from this systematic review. Non-English language articles and research conducted on non-human specimens were also excluded. This was followed by full-text screening for eligibility. The complete search strategy used is shown in Table 6. Table 7 depicts the inclusion and exclusion criteria for the articles.

The relevant studies were assessed with the Risk Of Bias In Non-randomized Studies-of Exposures (ROBINS-E) tool [78].

## 5. Conclusions

The study of the microbiome with next-generation sequencing allows more insight into the possible casual relationships between the bacteria and diseased state and not just culturable or cultivatable species. A unique and distinct microbial pattern could not be identified due to the vast heterogeneity present across all studies. The authors propose that future studies should investigate the microbial profile of peri-implantitis based on the severity of the disease to further provide insight into the progression and alteration of the microbial community within the peri-implant pocket.

A universal clinical parameter for the diagnosis of peri-implantitis should be implemented in all future studies to minimize the confounding factors. The subject pool should also be more diverse and larger to compensate for individual differences, and perhaps, a distinct microbial profile may be seen with a larger sample size. The studies reviewed also show that different groups of bacteria exist in the pockets at different stages of the diseases. This may imply that, with a complete microbial profile, an accurate estimation of the disease progression and monitoring can be performed. Furthermore, this also allows targeted drug therapies towards selective microorganisms that are strongly associated with peri-implantitis.

## Figures and Tables

**Table 1:** The Risk Of Bias In Non-randomized Studies–of Exposures (ROBINS-E) assessment.

Author, Year	Confounding Variables	Measurement of the Exposure	Selection of Participants	Post-Exposure Interventions	Missing Data	Measurement of Outcome	Selection of Reported Result	Overall Bias
Kim et al., 2023 [32]	S	L	S	S	L	L	L	S
Song et al., 2022 [33]	L	L	S	L	L	L	L	S
Pallos et al., 2022 [34]	H	L	L	L	L	L	L	H
Barbagallo et al., 2022 [35]	H	S	S	L	L	L	L	H
Shi et al., 2021 [36]	S	L	L	L	L	L	L	L
Polymeri et al., 2021 [37]	L	L	L	L	L	L	L	L
Korsch et al., 2021 [38]	L	L	L	L	L	L	L	L
Komatsu et al., 2020 [39]	S	L	L	L	L	L	L	L
Ghensi et al., 2020 [40]	S	S	S	L	L	L	L	S
Aleksandrowicz et al., 2020 [41]	S	L	L	L	L	L	L	L
Yu et al., 2019 [42]	S	L	L	L	L	L	L	L
Kröger et al., 2018 [43]	L	L	H	L	L	L	S	H
Gao et al., 2018 [44]	L	L	L	L	L	L	L	L
Daubert et al., 2018 [45]	L	S	L	L	L	L	L	S
Al-Ahmad et al., 2018 [46]	L	L	L	L	L	L	L	L
Sousa et al., 2016 [47]	L	L	L	L	L	L	L	L
Sanz-Martin et al., 2017 [20]	L	L	L	L	L	L	L	L
Apatzidou et al., 2017 [23]	S	S	L	L	L	L	L	S
Yu et al., 2016 [48]	S	L	L	L	L	L	L	L
Shiba et al., 2016 [49]	S	L	S	L	L	L	L	S
Tsigarida et al., 2015 [50]	L	L	L	L	L	L	L	L
Jakobi et al., 2015 [51]	S	L	S	L	L	L	L	S
Zheng et al., 2014 [52]	L	L	L	L	L	L	L	L
Schaumann et al., 2014 [53]	S	L	S	L	L	L	L	S
Maruyama et al., 2014 [54]	S	S	S	L	L	L	L	S
Tamura et al., 2013 [55]	L	L	L	L	L	L	L	L
Koyanagi et al., 2013 [21]	S	L	L	L	L	L	L	L
Dabdoub et al., 2013 [25]	L	L	L	L	L	L	L	L
da Silva et al., 2013 [56]	L	L	L	L	L	L	L	L
Kumar et al., 2012 [22]	H	S	S	L	L	L	L	H
Koyanagi et al., 2010 [57]	S	L	L	L	L	L	L	L
Faveri et al., 2010 [58]	L	L	L	L	L	L	L	L

L: low risk of bias; S: some concerns; H: high risk of bias.

**Table 2:** Grading of Recommendations, Assessment, Development, and Evaluation (GRADE) approach.

Certainty Assessment							Summary of Findings		
Participants (Studies) Follow-Up	Risk of Bias	Inconsistency	Indirectness	Imprecision	Publication Bias	Overall Certainty of Evidence	Study Event Rates (%)		Impact
							With Conventional Methods	With Next-Generation Sequencing	
**Outcome: Diversity and Richness**									
1069 (32 observational studies)	serious ^a^	serious ^b^	serious ^c^	not serious	All plausible residual confounding would reduce the demonstrated effect	Low	The diversity and richness of the microbiome is heterogeneous and inconsistent across all 32 studies.		
**Outcome: Abundance of Taxa**									
1069 (32 observational studies)	serious ^a^	serious ^b^	serious ^c^	not serious	All plausible residual confounding would suggest a spurious effect, while no effect was observed	Low	A heterogeneous pattern of taxa can be seen across all 32 studies reviewed.. The evidence suggests that next-generation sequencing has detected previously uncultured bacteria in diseased sites.		

^a^. Out of the 32 studies reviewed, nine were of some concern, while four were at a high risk of bias based on the ROBINS-E assessment tool. ^b^. Inconsistency is seen due to the heterogeneity across all 32 studies. ^c^. Indirectness is seen due to the differences in the severity of peri-implantitis. Microbial compositions of different severities present heterogenous results.

**Table 3:** Characteristics of the population and the results derived from the included studies.

Author, Year	Number of Subjects	Number of Implants	Study Setting	Duration of Implant	Case Definition for Peri-Implantitis/Peri-Implant Mucositis	Samples Collected	Collection Method
Kim et al., 2023 [32]	109	30 H, 30 PI	Korea	Not stated	PD ≥ 6 mm BOP Radiographic bone loss ≥3 mm	Supra- and subgingival plaque	Sterile Gracey curette
Song et al., 2022 [33]	14	14 H, 14 PI	China	Not stated	PD ≥ 6 mm Radiographic bone loss ≥3 mm	Subgingival plaque	Sterile paper point
Pallos et al., 2022 [34]	42	21 H, 21 PI	Brazil	≥2 years	PD ≥ 5 mm BOP ± suppuration Radiographic bone loss ≥3 mm	Unstimulated saliva	Sterile plastic tube
Barbagallo et al., 2022 [35]	24	10 H, 24 PI	Italy	≥1 year	Increasing PD since loading Evidence of radiographic bone loss BOP	Subgingival plaque	Sterile paper point
Shi et al., 2021 [36]	64	27 PM, 37 PI	China	≥1 year	PD ≥ 6 mm BOP/suppuration Marginal bone loss ≥3 mm	Subgingival plaque	Sterile paper point
Polymeri et al., 2021 [37]	41	41 PI	The Netherlands	≥1 year	PD ≥ 6 mm Clinical inflammation Radiographic bone loss ≥3 mm	Subgingival plaque	Sterile paper point
Korsch et al., 2021 [38]	48	31 PI, 22 H	Germany	≤3 months or ≥3 years	PD ≥ 6 mm BOP and suppuration Radiographic bone loss ≥6 mm	Subgingival plaque	Sterile paper point
Komatsu et al., 2020 [39]	21	21 PI	Japan	≥1 year	PD ≥ 6 mm BOP ± suppuration Radiographic bone loss ≥3 mm	Subgingival plaque	Sterile paper point
Ghensi et al., 2020 [40]	72	35 H, 37 PM, 41 PI	Italy	≥1 year	BOP Radiographic bone loss > 2 mm	Subgingival plaque	Sterile Gracey curette
Aleksandrowicz et al., 2020 [41]	139	37 H, 41 PI	Poland	Not stated	PD > 4 mm BOP Suppuration Visible three-thread loss	Subgingival plaque	Sterile Gracey curette
Yu et al., 2019 [42]	18	18 PI, 18 H	China	Not stated	PD ≥ 5 mm BOP and radiographic bone loss	Subgingival/submucosal plaque	Sterile paper point
Kröger et al., 2018 [43]	30	45 PI	Germany	Not stated	PD ≥ 5 mm BOP Radiographic bone loss ≥3 mm	Subgingival plaque	Sterile paper point
Gao et al., 2018 [44]	40	20 H, 20 PI	China	≥6 months	PD ≥ 4 mm BOP Radiographic bone loss ≥2 mm	Subgingival plaque	Sterile paper point
Daubert et al., 2018 [45]	9	5 H, 6 PI	USA	Not stated	PD ≥ 4 mm BOP ± suppuration Radiographic bone loss > 2 mm	Subgingival plaque	Sterile ½ mini Gracey curette
Al-Ahmad et al., 2018 [46]	10	10 H, 10 PI	Germany	Not stated	PD ≥ 5 mm BOP and radiographic bone loss	Subgingival plaque	Sterile paper point
Sousa et al., 2016 [47]	18	2 H, 2 PM, 2 PI	UK	Not stated	PD ≥ 5 mm Radiographic bone loss of more than three threads up to half of the implant length or ≥2.5 mm BOP	Subgingival plaque	Sterile Gracey curette
Sanz-Martin et al., 2017 [20]	67	35 PI, 32 H	Switzerland	≥1 year	Radiographic bone loss ≥2 mm at the mesial/distal side BOP	Subgingival plaque	Sterile Gracey curette
Apatzidou et al., 2017 [23]	10	4 H, 10 PI	Greece	≥1 year	PD ≥ 6 mm BOP/suppuration Radiographic bone loss ≥2 mm	Subgingival plaque	Sterile paper point
Yu et al., 2016 [48]	18	18 PI, 18 H	China	Not stated	PD ≥ 5 mm BOP and radiographic bone loss ≥2 mm	Subgingival plaque	Sterile paper point
Shiba et al., 2016 [49]	12	12 PI, 12 P	Japan	8.6 ± 7.2	PD ≥ 4 mm BOP and/or suppuration Radiographic bone loss	Subgingival plaque	Sterile paper point
Tsigarida et al., 2015 [50]	80	40 H, 20 PM, 20 PI	USA	≥4 years	Clinical inflammation (redness, swelling, BOP, suppuration) Radiographic bone loss > 2 mm	Subgingival plaque	Sterile paper point
Jakobi et al., 2015 [51]	18	9 H, 9 PI, 9 P	Germany	>6 months	Presence of mobility BOP ± suppuration	Subgingival plaque	Sterile paper point
Zheng et al., 2014 [52]	24	10 H, 8 PM, 6 PI	China	Not stated	Zitzmann & Berglundh (2008)	Subgingival plaque	Periodontal probe
Schaumann et al., 2014 [53]	7	4.7 ± 3.6 PI	Germany	≥1 year	PD ≥ 4 mm BOP Radiographic bone loss	Supra- and subgingival plaque	Sterile paper point
Maruyama et al., 2014 [54]	20	20 PI, 20 P	Japan	≥1 year	PD ≥ 4 mm BOP ± suppuration Presence of radiographic bone loss	Subgingival plaque	Sterile paper point
Tamura et al., 2013 [55]	30	15 H, 15 PI	Japan	>6 months	PD ≥ 4 mm BOP and suppuration Radiographic bone loss	Subgingival plaque	Sterile paper point
Koyanagi et al., 2013 [21]	6	6 PI	Japan	Not stated	PD ≥ 5 mm BOP and/or suppuration Radiographic bone loss of more than three threads up to half of the implant length	Subgingival plaque	Sterile paper point
Dabdoub et al., 2013 [25]	81	33 H, 20 PM, 20 PI	USA	≥1 year	Consensus Report of the Sixth European Workshop on Periodontology	Subgingival plaque	Sterile paper point
da Silva et al., 2013 [56]	20	10 PI, 20 H	Brazil	Not stated	PD ≥ 5 mm BOP and/or suppuration Saucer-shaped osseous defects of >3 mm	Subgingival plaque	Sterile Gracey curette
Kumar et al., 2012 [22]	40	10 H, 10 PI	USA	≥1 year	Classification of Periodontal Diseases (Armitage 1999) Consensus Report on Peri-Implant Diseases (Lindhe & Meyle 2008)	Subgingival plaque	Sterile paper point
Koyanagi et al., 2010 [57]	3	3 H, 3 PI	Japan	3–10	PD ≥ 5 mm BOP and/or suppuration Radiographic bone loss of more than three threads up to half of the implant length	Subgingival plaque	Sterile paper point
Faveri et al., 2010 [58]	50	25 H, 25 PI	Brazil	Not stated	PD ≥ 5 mm Saucer-shaped osseous defects of >3 mm BOP and/or suppuration	Subgingival plaque	Sterile Gracey curette

PD: probing depth; BOP: bleeding on probing; P: periodontitis; PI: peri-implantitis; H: healthy implant; PM: peri-implant mucositis.

**Table 4:** Summary of techniques of DNA extraction, amplification, and sequencing.

Author, Year	Method of DNA Extraction	DNA Amplification and Targeted Region	Sequencing Technique	Reference Database
Kim et al., 2023 [32]	Lucigen DNA kit, LGC Biosearch Technologies, Middleton, USA	PCR amplification of the 16s rRNA gene at the V3–V4 region	Illumina MiSeq	Human Oral Microbiome Database
Song et al., 2022 [33]	TIANamp Micro DNA Isolation Kit, TIANGEN BIOTECH, Beijing, China	PCR amplification at the V3–V4 hypervariable region of 16S rRNA with the primers 338F and 806R	Illumina MiSeq	Human Oral Microbiome database
Pallos et al., 2022 [34]	NucliSENS easyMAG, bioMérieux, Missouri, USA	V4 hypervariable region of the 16S rRNA gene was amplified using F515 and R80	Ion 318™ Chip kit v2 400-base chemistry	HOMD and Greengene amd NCBI 16s rRNA reference sequence
Barbagallo et al., 2022 [35]	PureLink Genomic DNA kit, Thermo Fisher Scientific, USA	PCR amplification of the 16s rRNA gene at V3–V4 region	Illumina Miseq	Human Oral Microbiome database
Shi et al., 2021 [36]	DNeasy PowerSoil kit, QIAGEN, Venlo, The Netherlands	PCR amplification of the 16S rRNA genes at V3–V4 region	Illumina MiSeq	Silva database
Polymeri et al., 2021 [37]	AGOWA mag Mini DNA Isolation Kit, LGC Genomics, Teddington, United Kingdom	PCR amplification of the 16S rRNA gene hypervariable region V5–V7.	454 GS-FLX + Titanium system was used for pyrosequencing	Ribosomal Database Project & Human Oral Microbiome Database
Korsch et al., 2021 [38]	Qiagen DNA MiniAmp Kit, QIAGEN, Venlo, The Netherlands	PCR amplification of the 16s rRNA gene at V1–V2 region	Illumina MiSeq	Silva database
Komatsu et al., 2020 [39]	Mora-extract, AMR Inc., Tokyo, Japan	Not stated	Illumina Miseq	Human Oral Microbiome database
Ghensi et al., 2020 [40]	Qiagen DNA MiniAmp kit, QIAGEN, Venlo, The Netherlands	Not stated	Illumina Hiseq	MetaPhlAn 2 and HUMAnN2
Aleksandrowicz et al., 2020 [41]	Genomic Mini kit, A&A Biotechnology, Gdańsk, Poland	The 2720 Thermal Cycler was used for the amplification of archaeal and bacterial DNA. Oligonucleotide-specific primers were used to target the specific 16s rRNA gene	3130xl Genetic Analyzer	GenBank
Yu et al., 2019 [42]	Qiagen DNA MiniAmp kit, QIAGEN, Venlo, The Netherlands	PCR amplification at the hypervariable region V3–V4 of 16s rRNA	Paired-end MiSeq sequencing	Human Oral Microbiome Database
Kröger et al., 2018 [43]	Sigma-Aldrich GenElute Bacterial Genomic DNA Kit, Sigma-Aldrich, Munich, Germany	PCR amplification of the 16s rRNA gene at V3–V4 regions	Illumina MiSeq	Human Oral Microbiome Database
Gao et al., 2018 [44]	Not stated	PCR amplification of the 16S V3–V4 regions with primers 343F and 798R	Illumina Miseq	Human Oral Microbiome database
Daubert et al., 2018 [45]	Chelex-100, Bio-Rad, Hercules, USA	PCR amplification was used to amplify prokaryotic 16S rRNA genes using universal primers (27F and 1392R). Region of amplification not stated	Roche 454	Human Oral Microbiome database
Al-Ahmad et al., 2018 [46]	DNeasy Blood and Tissue kit, QIAGEN, Venlo, The Netherlands	PCR amplification of 16s rRNA using the universal primers 27F-YM and 1492R, region not stated	Ridom TraceEdit software, version 1.1.0	GenBank
Sousa et al., 2016 [47]	Not stated	Amplification with PCR using the 16S rRNA gene with V5–V7 primers	Illumina MiSeq	Greengenes
Sanz-Martin et al., 2017 [20]	Masterpure purification kit, Epicentre, Wisconsin, USA	PCR amplification of the 16s rRNA gene at V3–V4 region	Illumina MiSeq	Ribosomal Database Project (RDP)
Apatzidou et al., 2017 [23]	Proteinase K (100 mcg/mL) at 60 °C for 60 min, later boiled for 10 min Concentration measured with the Nanodrop NP-1000 spectrophotometer (Thermo Fisher Scientific, Renfrew, UK) Final concentration adjusted to 5 ng/mcL	PCR amplification of the V3–V4 region of the 16s rRNA gene	Illumina MiSeq	Greengenes database
Yu et al., 2016 [48]	Qiagen DNA MiniAmp kit, QIAGEN, Venlo, The Netherlands	PCR amplification of 16s rRNA at ca. 650 bp regions corresponding to the V2–V5 region	M13 forward primer	Human Oral Microbiome Database
Shiba et al., 2016 [49]	Not stated	PCR amplification of 16s rRNA, region not stated	Illumina MiSeq	Human Oral Microbiome Database
Tsigarida et al., 2015 [50]	Qiagen DNA MiniAmp kit, QIAGEN, Venlo, The Netherlands	PCR amplification of the V1 to V3 and V7 to V9 regions	The TTitanium platform was used to perform multiplexed bacterial-tag-encoded FLX amplicon pyrosequencing.	Human Oral Microbiome Database
Jakobi et al., 2015 [51]	Qiagen DNA MiniAmp kit, QIAGEN, Venlo, The Netherlands	PCR amplification of 16s rDNA	Not stated	Ribosomal Database Project
Zheng et al., 2014 [52]	Not stated	PCR was used to amplify the V1–V3 regions of the 16s rRNA gene	The 454-GS-FLX sequencing platform was used for pyrosequencing	Ribosomal Database Project
Schaumann et al., 2014 [53]	QIAamp DNA MiniAmp Kit, QIAGEN, Venlo, The Netherlands	PCR amplification of 16s rRNA at the V1–V3 regions	Pyrosequencing was performed via the GS FLX sequencer	Greengenes
Maruyama et al., 2014 [54]	Mora-extract, AMR Inc. Tokyo, Japan	PCR amplification of the 16S V3–V4 regions with primers 806R and 515F	Roche 454	Ribosomal Database Project, Human Oral Microbiome Database, and NCBI
Tamura et al., 2013 [55]	Not stated	PCR amplification of the 16s rRNA gene with the forward primers 16S27F and 16S341F and the reverse primers 16S1492R and 16S907R	Takara Bio	GenBank database
Koyanagi et al., 2013 [21]	Mora-extract, AMR Inc. Tokyo, Japan	PCR amplification of the 16s rRNA gene with the primers 27F and 1492R	The 27F and 520R primers (BigDye Terminator Cycle Sequencing kit) were used, and 3130xl Genetic Analyzer	Ribosomal Database Project-II (RDP-II)
Dabdoub et al., 2013 [25]	Qiagen DNA MiniAmp kit, QIAGEN, Venlo, The Netherlands	PCR amplification of the 16s rRNA gene at two regions: V1–V3 and V7–V9	Pyrotag sequencing was performed	Greengenes
da Silva et al., 2013 [56]	Masterpure DNA purification kit, Epicentre, Wisconsin, USA	Two step PCR was performed. The first step involved two sets of forward primers in a 1:1 ratio and the reverse primer 1541R. The second step involved the same two sets of forward primers and the reverse primer 1492R.	ABI Prism fluorescent bases	Ribosomal Data Project (RDP) & GenBank
Kumar et al., 2012 [22]	Qiagen DNA MiniAmp kit, QIAGEN, Venlo, The Netherlands	PCR amplification of 16s rRNA at the V1–V3 and V7–V9 regions	The Titanium platform was used to perform multiplexed bacterial-tag-encoded FLX amplicon pyrosequencing.	Greengenes
Koyanagi et al., 2010 [57]	Mora-extract, AMR Inc. Tokyo, Japan	PCR amplification of plasmid DNA	27F and 520R primers (BigDye Terminator Cycle Sequencing kit) were used and the 3130xl Genetic Analyzer	Ribosomal Database Project-II (RDP-II)
Faveri et al., 2010 [58]	Proteinase K (200 mg/mL) was added to the buffer and then inactivated at 95 °C	PCR amplification with the universal primer pair for Euryarchaea and the reverse primer 954rEyAr	ABI Prism fluorescent bases	Ribosomal Data Project (RDP) & GenBank

PCR: Polymerase chain reaction.

**Table 5:** Microbial profiles from the retrieved studies showing the diversity and richness and the abundance of taxa.

Author, Year	Groups	Results	
		Diversity and Richness	Abundance of Taxa
Kim et al., 2023 [32]	Peri-implantitis Periodontitis	PI = P ^a^ PI > P ^b^	PI&P: *P. gingivalis*, *Prevotella* spp., *Treponema* spp., *F. alocis*, and *F. fastidiosum* PI > P: *Anaerotignum lactatifermentans*, *Bacteroides vulgatus*, *Faecalibacterium prausnitzii*, *Olsenella uli*, *Parasutterella excrementihominis*, *Prevotella buccae*, *P. alactolyticus*, and *Slackia exigua*
Song et al., 2022 [33]	Peri-implantitis	PI = HI ^b^ PI > HI ^e^ HI ≠ PI ^c^ (Significant difference between groups)	PI: *Bacteroidetes*, *Spirochaetes*, *and Synergistetes*, *as well as the genera of Porphyromonas*, *Treponema*, *Filifactor*, *Fretibacterium*, *Lachnospiraceae G-8*, and *Peptostreptococcaceae XIG-1* HI: *Proteobacteria*, *Neisseria*, *Streptococcus*, *Haemophilus*, and *Rothia*
Pallos et al., 2022 [34]	Peri-implantitis	HI > PI ^a,e^ HI = PI ^c^	PI > HI: *Stenotrophomonas*, *Enterococcus*, *Leuconostoc genus*, *Faecalibacterium prausnitzii*, *Haemophilus parainfluenzae*, *Prevotella copri*, *Bacteroides vulgatus*, and *Bacteroides stercoris*
Barbagallo et al., 2022 [35]	Peri-implantitis Periodontitis	PI > P ^a^ PI = P ^b^	PI: *Peptostreptococcaceae*, *Dialister*, *Mongibacterium*, *Atopobium*, and *Filifactor* P: *Bacteroidales*
Shi et al., 2021 [36]	Peri-implantitis Peri-implant mucositis	PI = PM (No significant difference between groups) ^a,b,c^	PI = PM: No significant difference, *Bacteroidetes* (45.08% in PM, 42.89% in PI), *Firmicutes* (21.03% in PM, 19.44% in PI), *Proteobacteria* (11.16% in PM, 10.41% in PI) *Fusobacteria* (11.12% in PM, 14.7% in PI), *Spirochetes* (8.38% in PM, 9.68% in PI), *Porphyromonas* (17.04% in PM, 16.54% in PI), *Fusobacterium* (9.78% in PM, 12.39% in PI), *Treponema* (8.37% in PM, 9.59% in PI) and *Prevotella* (7.43% in PM, 7.04% in PI). PI > PM: *Holdemanella* and *Cardiobacterium* PM > PI: *Oribacterium*, *Staphylococcus*, and *Ramlibacter*
Polymeri et al., 2021 [37]	Peri-implantitis Peri-implant mucositis	HI = PM = PI (No significant differences between groups) ^a,b,g^	PI: *Fusobacterium nucleatum* and *Treponema denticola* PM: *Rothia mucilaginosa* and *Streptococcus salivarius*
Korsch et al., 2021 [38]	Peri-implantitis	PI > HI ^d^	PI: *Fusobacterium nucleatum* and *Porphyromonas gingivalis* HI: *Streptococcus*, *Neisseria*, *Rothia* and *Veillonella*
Komatsu et al., 2020 [39]	Peri-implantitis Periodontitis	PI > P ^a^ PI = P ^c,g^	PI: *Solobacterium moorei* and *Prevotella denticola* P: *F. nucleatum*, *P. stomatis* and *Leptotrichia* sp.
Ghensi et al., 2020 [40]	Peri-implantitis Peri-implant mucositis	PI < HI ^a,b^	PI: *Treponema maltophilum*, *Fretibacterium fastidiosum*, *Pseudoramibacter alactolyticus*, *T. lecithinolyticum*, *P. gingivalis*, *T. forsythia*, *Treponema denticola*, *P. endodontalis*, *Filifactor alocis*, and *Desulfobulbus* spp. HI: *C. gingivalis*, *C. granulosa*, *C. ochracea*, *S. noxia*, *S. artemidis*, *Actinomyces*, *Capnocytophaga*, *Neisseria*, *Rothia*, and *Streptococcus*
Aleksandrowicz et al., 2020 [41]	Peri-implantitis Periodontitis	Nil	PI: *F nucleatum* and *T denticola*
Yu et al., 2019 [42]	Peri-implantitis Periodontitis	PI = HI (No significant difference between groups) ^d,f^	PI=HI: *Streptococcus infantis/mitis/oralis* (*HMT-070/HMT-071/HMT-638/HMT-677*) and *Fusobacterium* sp. *HMT-203/HMT-698* PI (Low abundance): *Aquificae*, *Chlamydiae*, *Gemmatimonadetes*, *Nitrospirae*, *TM6*, *Verrucomicrobia*, and *WPS2* phyla
Kröger et al., 2018 [43]	Peri-implantitis	PI > HI ^g^	PI: *Eubacteriaceae* [XV], *Fretibacterium* sp. *HMT 362*, *Fretibacterium fastidiosum*, *Peptostreptococcaceae* [XI][G-6], *Alloprevotella* sp. *HMT 473*, *Fastidiosipila sanguinis*, *Filifactor alocis*, *Peptostreptococcaceae* [XI][G-4], *Bacteriodetes* [G-3] bacterium *HMT 365*, *Treponema parvum*, *Clostridiales* [F-1][G-1] bacterium *HMT 093*, and *Orobacterium*
Gao et al., 2018 [44]	Peri-implantitis	PI > HI ^b^ HI ≠ PI (Significant difference between groups) ^c^	PI: *Moraxella*, *Micrococcus*, and *Acinetobacter* HI: *Neisseria*, *Haemophilus*, *Prevotella*, *Streptococcus*, *Porphyromonas*, *Clostridium*, *Capnocytophaga*, *Leptothrix*, *Actinomycetes*, and *Actinomyces*
Daubert et al., 2018 [45]	Peri-implantitis	HI > PI ^a,b,c^	PI: *Veillonella* and *Neisseria*.
Al-Ahmad et al., 2018 [46]	Peri-implantitis	Not reported	PI: *Bacteroidetes* (phylum), *Fusobacterium nucleatum*
Sousa et al., 2016 [47]	Peri-implantitis Aggressive periodontitis Peri-implant mucositis	P > PI ^a,b,f^	PI: *Propionibacterium*, *Paludibacterium*, *Staphylococcus*, *Filifactor*, *Mogibacterium*, *Bradyrhizobium*, and *Acinetobacter*
Sanz-Martin et al., 2017 [20]	Peri-implantitis	PI > HI ^c^	PI: *Bacteroides*, *Spirochetes*, and *Synergistetes*, *Tannerella forsythia*, *Treponema denticola*, and *Porphyromonas gingivalis*, *Filifactor alocis*, *Fretibacterium fastidiosum*, and *Treponema maltophilum* HI: *Proteobacteria* and *Actinobacteria* PI > HI: *Porphyromonas* (phylum *Bacteroidetes*), *Treponema* (phylum *Spirochetes*), *Filifactor* (phylum *Firmicutes*), *Fretibacterium* (phylum *Synergistetes*), *Tannerella* (phylum *Bacteroidetes*), *T. forsythia*, *P. gingivalis*, and *T. denticola*). HI > PI: *Streptococcus* (phylum *Firmicutes*), *Veillonella* (phylum *Firmicutes*), *Rothia* (phylum *Actinobacteria*), *Haemophilus* (phylum *Proteobacteria*) and *Neisseria* spp.
Apatzidou et al., 2017 [23]	Peri-implantitis	PI > HI ^a^ HI = PI (No significant difference between groups) ^b^	HI: *Actinobacillus* and *Streptococcus* PI: *Prevotella*, *Porphyromonas*, *Synergistetes*
Yu et al., 2016 [48]	Peri-implantitis Periodontitis	PI ≠ HI (Significant difference between groups) ^f^	PI: High abundance of *F. fastidiosum* and *Fretibacterium*
Shiba et al., 2016 [49]	Peri-implantitis Smoking Periodontitis	PI = P (No significant difference between groups) ^a,g^ PI ≠ P (Significant difference between groups) ^c^	PI = P: High rc-rRNA abundances *Porphyromonas gingivalis*, *Treponema denticola*, and *Tannerella forsythia*
Tsigarida et al., 2015 [50]	Peri-implantitis Smoking Peri-implant mucositis	HI = PI ^b^ HI ≠ PI (Significant difference between groups) ^c^	PI: *Aggregatibacter*, *Capnocytophaga*, *Corynebacterium mucifaciens*, *Fretibacterium*, *Lachnoanaerobaculum*, *Lactobacillus panis*, *Neisseria*, *Prevotella* HI: *Actinomyces*, *Alloprevotella*, *Capnocytophaga*, *Enterobacter cancerogenus*, *Fusobacterium gonidiaformans*, *Fusobacterium*, *Lactobacillus johnsonii*, *Neisseria lactamica*, *Porphyromonas asaccharolytica*, *Prevotella enoeca*, *Prevotella*, *Pseudomonas*, *Pseudomonas pseudoalcaligenes*, *SR1 [G-1]*, *Streptococcus*, *Tannerella*
Jakobi et al., 2015 [51]	Peri-implantitis Periodontitis	Not reported	PI and P: *Enterococcus*, *Streptococcus*, *Porphyromonas*, *Fusobacterium*, *Prevotella*, *Bacillus*, and *Fretibacterium* Exclusive to PI: *Neisseria* and *Kingella* Exclusive to P: *Tannerella*, *Rothia*, *Parabacteroides*, *Parvimonas*, and *Filifactor* HI: *Enterococcus*, *Bacillus*, *Streptococcus*, *Fusobacterium*, *Prevotella*, *Porphyromonas*, *Rothia* and *Proteus*
Zheng et al., 2014 [52]	Peri-implantitis Peri-implant mucositis	PM = PI (No significant differences among groups) ^f^ HI > PM ^f^ HI > PI ^f^ PI > HI ^a,b,g^	PI: *Leptotrichia hofstadii*, *Eubacterium infirmum*, *Kingella denitrificans*, *Actinomyces cardiffensis*, *Eubacterium minutum*, *Treponema lecithinolyticum*, and *Gemella* sanguinis, *Gemella sanguinis*, *Eubacterium minutum*, and *Actinomyces cardiffensis*
Schaumann et al., 2014 [53]	Peri-implantitis Periodontitis	PI = P (No significant difference between groups) ^a^	PI: *Porphyromonadaceae*, *Lachnospiraceae*, and *Streptococcaceae;* Genera *Rothia*, *Actinomyces*, *Paenibacillus*, *Microbacterium*, *Pseudoramibacter*, *Leptotrichia*, *Parascardovia*, *Tannerella*, *Granulicatella*, *Tessaracoccus*, *Clostridium*, *Aeromonadales*, *Veillonella*, *Capnocytophaga*, *Prevotella*, *TG5*, *Fusobacterium*, *Exiguobacterium*, *Enterococcus*, *Porphyromonas* and *Streptococcus*.
Maruyama et al., 2014 [54]	Peri-implantitis Periodontitis	PI = P ^a,b,c,g^ (no significant difference)	PI: *Prevotella nigrescens*, *Olsenella*, *Sphingomonas*, *Peptostreptococcus*, and *Neisseriaceae* P: *Peptostreptococcaceae* sp. and *Desulfomicrobium orale*
Tamura et al., 2013 [55]	Peri-implantitis	Not reported	PI: *E nodatum*, *P intermedia*, *F nucleatum*, *Filifactor alocis*, *E brachy*, *Parascardovia denticolens*, *Parvimonas micra* HI: *Veillonella* sp., *Propionibacterium acnes*, *Pseudoramibacter alactolyticus*, *Parvimonas micra*
Koyanagi et al., 2013 [21]	Peri-implantitis Periodontitis	PI > P ^a,b^	PI and P: *Firmicutes* and *Bacteroidetes*, *Fusobacterium* spp. and *Streptococcus* spp., Exclusive to PI: *Parvimonas micra*, *Peptostreptococcus stomatis*, *Pseudoramibacter alactolyticus*, and *Solobacterium moorei* PI > P sites: *Dialister* spp., *Eubacterium* spp., *Porphyromonas* spp., *P. gingivalis.* PI = P sites: *T. forsythia*, *T. denticola*
Dabdoub et al., 2013 [25]	Peri-implantitis Periodontitis	P > PI ^a^	PI = P: No significant difference in the number of shared species
da Silva et al., 2013 [56]	Peri-implantitis	Not reported	HI: *Actinomyces*, *Atopobium*, *Gemella*, *Kingella* and *Rothia*, *Campylobacter*, *Desulfobulbus*, *Dialister*, *Eubacterium*, *Filifactor*, *Mitsukella*, *Porphyromonas* and *Pseudoramibacter*. PI > HI: *Fusobacterium nucleatum*, *Dialister invisus*, *Streptococcus* sp. human oral taxon (*HOT*) *064*, *Filifactor alocis*, and *Mitsuokella* sp. *HOT 131* HI > PI: *Veillonella dispar*, *Actinomyces meyeri*, and *Granulicatella adiacens*
Kumar et al., 2012 [22]	Peri-implantitis Periodontitis	HI > PI ^c^ P > PI ^a^	PI: *Actinomyces*, *Peptococcus*, *Campylobacter*, *nonmutans Streptococcus*, *Butyrivibrio*, and *Streptococcus mutans*, *B. fibrisolvens*
Koyanagi et al., 2010 [57]	Peri-implantitis Periodontitis	PI > P ^a,b^	PI: *Chloroflexi*, *Tenericutes*, and *Synergistetes* phyla Exclusive to PI: *Parvimonas* micra, *Peptostreptococcus stomatis*, *Pseudoramibacter alactolyticus*, *Fusobacterium nucleatum*, and *Solobacterium moorei* Detected in P: *Fusobacterium nucleatum*, *Granulicatella adiacens*
Faveri et al., 2010 [58]	Peri-implantitis	Not reported	PI: *Archaea* detected at significantly higher abundance

PI: Peri-implantitis; HI: healthy implants; P: periodontitis; PM: peri-mucositis. ^a^: Shannon’s index; ^b^: Chao1 index; ^c^: Principal Coordinate Analysis (PCoA); ^d^: permutational multivariate analysis of variance (PERMANOVA); ^e^: InvSimpson’s index; ^f^: weighted Unifrac distance analysis; ^g^: number of operational taxonomic units (OTUs).

**Table 6:** Search strategies employed.

Database	Search Terms
Medline	(Peri-implantiti$ OR Peri adj2 Implantiti$ OR Peri-implant$ adj2 inflam$ OR Peri-implant$ adj2 infect$ OR Peri-implant$ adj2 disease$ OR exp Peri-Implantitis/or exp Dental Implants/or exp Dental Implantation, Endosseous/OR peri-implant adj2 mucositi$ OR peri adj2 implant adj2 mucositi$ OR periimplant adj2 mucositi$ OR periimplant$ adj2 mucos$) AND (exp sequence analysis/or exp sequence analysis, dna/or exp sequence analysis, rna/or exp rna-seq/OR exp RNA, Ribosomal, 16S/OR exp Microbiota/OR exp Bacteria/)
Cochrane	(peri-implantiti* OR periimplantiti* OR (Peri-Implantitis):ti,ab,kw OR Peri-implant* NEAR/2 inflam* OR Peri-implant* NEAR/2 infect* OR peri-implant muco*sitis OR peri-implant NEAR/2 disease* OR peri-implant infect* OR MeSH descriptor: [Peri-Implantitis] explode all trees OR periimplant* NEAR/2 mucos*) AND (dental implant* OR dental implant, endosseous OR endosseous dental implant*) AND (MeSH descriptor: [Sequence Analysis, DNA] explode all trees OR MeSH descriptor: [Sequence Analysis] explode all trees OR MeSH descriptor: [Sequence Analysis, RNA] explode all trees OR MeSH descriptor: [RNA-Seq] explode all trees OR MeSH descriptor: [RNA, Ribosomal, 16S] explode all trees OR MeSH descriptor: [Microbiota] explode all trees OR MeSH descriptor: [Bacteria] explode all trees)
Scopus	(peri-implant* OR peri W/2 implant* OR peri-implant* W/2 inflam* OR peri-implant* W/2 infect* OR peri-implant* W/2 disease* OR peri-implant W/2 mucositi* OR peri W/2 implant W/2 mucositi* OR periimplant W/2 mucositi* OR periimplant* W/2 mucos*) AND (dental AND implants OR dental AND implantation AND endosseous) AND ((sequence AND analysis) OR (sequence AND analysis AND dna) OR (sequence AND analysis AND rna) OR rna-seq OR (rna AND ribosomal AND 16s)) AND (microbiota OR bacteria)

ti: Title; ab: Abstract; kw: Keywords; exp: Explode.

**Table 7:** Inclusion and exclusion criteria used for the studies screened.

Inclusion Criteria	Exclusion Criteria
Observational and case-control studies investigating the microbiome of peri-implant tissues through next-generation DNA sequencing methods. Human studies in English	Culture-based studies, conference papers, review articles, studies regarding peri-implantitis associated with other systematic factors (diabetes mellitus, immune disorders, etc.) Articles that examined only specific microorganisms. Non-English language articles and research conducted on non-human specimens.

## Data Availability

All data are provided with the manuscript.

## References

[1] Buser D., Janner S.F.M., Wittneben J.-G., Brägger U., Ramseier C.A., Salvi G.E. (2012). 10-Year Survival and Success Rates of 511 Titanium Implants with a Sandblasted and Acid-Etched Surface: A Retrospective Study in 303 Partially Edentulous Patients: 10-Year Survival and Success Rates of SLA Implants. Clin. Implant Dent. Relat. Res..

[2] Serino G., Hultin K. (2019). Periimplant Disease and Prosthetic Risk Indicators: A Literature Review. Implant Dent..

[3] Pesce P., Canullo L., Grusovin M.G., de Bruyn H., Cosyn J., Pera P. (2015). Systematic Review of Some Prosthetic Risk Factors for Periimplantitis. J. Prosthet. Dent..

[4] Derks J., Tomasi C. (2015). Peri-Implant Health and Disease. A Systematic Review of Current Epidemiology. J. Clin. Periodontol..

[5] Rodrigo D., Sanz-Sánchez I., Figuero E., Llodrá J.C., Bravo M., Caffesse R.G., Vallcorba N., Guerrero A., Herrera D. (2018). Prevalence and Risk Indicators of Peri-Implant Diseases in Spain. J. Clin. Periodontol..

[6] Roos-Jansåker A.-M., Lindahl C., Renvert H., Renvert S. (2006). Nine- to Fourteen-Year Follow-up of Implant Treatment. Part II: Presence of Peri-Implant Lesions. J. Clin. Periodontol..

[7] Fransson C., Lekholm U., Jemt T., Berglundh T. (2005). Prevalence of Subjects with Progressive Bone Loss at Implants: Prevalence of Subjects with Progressive Bone Loss at Implants. Clin. Oral Implant Res..

[8] Diaz P., Gonzalo E., Villagra L.J.G., Miegimolle B., Suarez M.J. (2022). What Is the Prevalence of Peri-Implantitis? A Systematic Review and Meta-Analysis. BMC Oral Health.

[9] Astolfi V., Ríos-Carrasco B., Gil-Mur F.J., Ríos-Santos J.V., Bullón B., Herrero-Climent M., Bullón P. (2022). Incidence of Peri-Implantitis and Relationship with Different Conditions: A Retrospective Study. Int. J. Environ. Res. Public Health.

[10] Hashim D., Cionca N. (2020). A Comprehensive Review of Peri-Implantitis Risk Factors. Curr. Oral Health Rep..

[11] Kumar P.S. (2019). Systemic Risk Factors for the Development of Periimplant Diseases. Implant Dent..

[12] Steiger-Ronay V., Merlini A., Wiedemeier D.B., Schmidlin P.R., Attin T., Sahrmann P. (2017). Location of Unaccessible Implant Surface Areas during Debridement in Simulated Peri-Implantitis Therapy. BMC Oral Health.

[13] Apatzidou D.A., Kinane D.F., Mombelli A. (2011). Modern Approaches to Non-Surgical Biofilm Management. Frontiers of Oral Biology.

[14] James P., Worthington H.V., Parnell C., Harding M., Lamont T., Cheung A., Whelton H., Riley P. (2017). Chlorhexidine Mouthrinse as an Adjunctive Treatment for Gingival Health. Cochrane Database Syst. Rev..

[15] Lombardo G., Signoriello A., Corrocher G., Signoretto C., Burlacchini G., Pardo A., Nocini P.F. (2019). A Topical Desiccant Agent in Association with Manual Debridement in the Initial Treatment of Peri-Implant Mucositis: A Clinical and Microbiological Pilot Study. Antibiotics.

[16] Herrera D., Berglundh T., Schwarz F., Chapple I., Jepsen S., Sculean A., Kebschull M., Papapanou P.N., Tonetti M.S., Sanz M. (2023). Prevention and Treatment of Peri-Implant Diseases—The EFP S3 Level Clinical Practice Guideline. J. Clin. Periodontol..

[17] Ferreira S.D., Martins C.C., Amaral S.A., Vieira T.R., Albuquerque B.N., Cota L.O.M., Esteves Lima R.P., Costa F.O. (2018). Periodontitis as a Risk Factor for Peri-Implantitis: Systematic Review and Meta-Analysis of Observational Studies. J. Dent..

[18] Renvert S., Lindahl C., Renvert H., Persson G.R. (2008). Clinical and Microbiological Analysis of Subjects Treated with Brånemark or AstraTech Implants: A 7-Year Follow-up Study. Clin. Oral Implant Res..

[19] Salvi G.E., Fürst M.M., Lang N.P., Persson G.R. (2008). One-Year Bacterial Colonization Patterns of Staphylococcus Aureus and Other Bacteria at Implants and Adjacent Teeth. Clin. Oral Implant Res..

[20] Sanz-Martin I., Doolittle-Hall J., Teles R.P., Patel M., Belibasakis G.N., Hämmerle C.H.F., Jung R.E., Teles F.R.F. (2017). Exploring the Microbiome of Healthy and Diseased Peri-Implant Sites Using Illumina Sequencing. J. Clin. Periodontol..

[21] Koyanagi T., Sakamoto M., Takeuchi Y., Maruyama N., Ohkuma M., Izumi Y. (2013). Comprehensive Microbiological Findings in Peri-Implantitis and Periodontitis. J. Clin. Periodontol..

[22] Kumar P.S., Mason M.R., Brooker M.R., O’Brien K. (2012). Pyrosequencing Reveals Unique Microbial Signatures Associated with Healthy and Failing Dental Implants. J. Clin. Periodontol..

[23] Apatzidou D., Lappin D.F., Hamilton G., Papadopoulos C.A., Konstantinidis A., Riggio M.P. (2017). Microbiome of Peri-Implantitis Affected and Healthy Dental Sites in Patients with a History of Chronic Periodontitis. Arch. Oral Biol..

[24] Rakic M., Grusovin M.G., Canullo L. (2016). The Microbiologic Profile Associated with Peri-Implantitis in Humans: A Systematic Review. Int. J. Oral Maxillofac. Implant..

[25] Dabdoub S.M., Tsigarida A.A., Kumar P.S. (2013). Patient-Specific Analysis of Periodontal and Peri-Implant Microbiomes. J. Dent. Res..

[26] Heuer W., Kettenring A., Stumpp S.N., Eberhard J., Gellermann E., Winkel A., Stiesch M. (2012). Metagenomic Analysis of the Peri-Implant and Periodontal Microflora in Patients with Clinical Signs of Gingivitis or Mucositis. Clin. Oral Investig..

[27] Mombelli A., Décaillet F. (2011). The Characteristics of Biofilms in Peri-Implant Disease. J. Clin. Periodontol..

[28] Mombelli A., van Oosten M.A., Schurch E., Land N.P. (1987). The Microbiota Associated with Successful or Failing Osseointegrated Titanium Implants. Oral Microbiol. Immunol..

[29] Qin D. (2019). Next-Generation Sequencing and Its Clinical Application. Cancer Biol. Med..

[30] Chen P., Sun W., He Y. (2020). Comparison of the Next-Generation Sequencing (NGS) Technology with Culture Methods in the Diagnosis of Bacterial and Fungal Infections. J. Thorac. Dis..

[31] Torchia M.T., Austin D.C., Kunkel S.T., Dwyer K.W., Moschetti W.E. (2019). Next-Generation Sequencing vs Culture-Based Methods for Diagnosing Periprosthetic Joint Infection After Total Knee Arthroplasty: A Cost-Effectiveness Analysis. J. Arthroplast..

[32] Kim H.-J., Ahn D.-H., Yu Y., Han H., Kim S.Y., Joo J.-Y., Chung J., Na H.S., Lee J.-Y. (2023). Microbial Profiling of Peri-Implantitis Compared to the Periodontal Microbiota in Health and Disease Using 16S rRNA Sequencing. J. Periodontal Implant Sci..

[33] Song L., Jiang J., Li J., Zhou C., Chen Y., Lu H., He F. (2022). The Characteristics of Microbiome and Cytokines in Healthy Implants and Peri-Implantitis of the Same Individuals. J. Clin. Med..

[34] Pallos D., Sousa V., Feres M., Retamal-Valdes B., Chen T., Curtis M., Boaventura R.M., Tanaka M.H., Salomão G.V.D.S., Zanella L. (2022). Salivary Microbial Dysbiosis Is Associated With Peri-Implantitis: A Case-Control Study in a Brazilian Population. Front. Cell. Infect. Microbiol..

[35] Barbagallo G., Santagati M., Guni A., Torrisi P., Spitale A., Stefani S., Ferlito S., Nibali L. (2022). Microbiome Differences in Periodontal, Peri-Implant, and Healthy Sites: A Cross-Sectional Pilot Study. Clin. Oral Investig..

[36] Shi Y., Tong Z., Zhang Y., Si M., He F. (2022). Microbial Profiles of Peri-implant Mucositis and Peri-implantitis: Submucosal Microbial Dysbiosis Correlates with Disease Severity. Clin. Oral Implant. Res..

[37] Polymeri A., Horst J., Buijs M.J., Zaura E., Wismeijer D., Crielaard W., Loos B.G., Laine M.L., Brandt B.W. (2021). Submucosal Microbiome of Peri-implant Sites: A Cross-sectional Study. J. Clin. Periodontol..

[38] Korsch M., Marten S.-M., Stoll D., Prechtl C., Dötsch A. (2021). Microbiological Findings in Early and Late Implant Loss: An Observational Clinical Case-Controlled Study. BMC Oral Health.

[39] Komatsu K., Shiba T., Takeuchi Y., Watanabe T., Koyanagi T., Nemoto T., Shimogishi M., Shibasaki M., Katagiri S., Kasugai S. (2020). Discriminating Microbial Community Structure Between Peri-Implantitis and Periodontitis With Integrated Metagenomic, Metatranscriptomic, and Network Analysis. Front. Cell. Infect. Microbiol..

[40] Ghensi P., Manghi P., Zolfo M., Armanini F., Pasolli E., Bolzan M., Bertelle A., Dell’Acqua F., Dellasega E., Waldner R. (2020). Strong Oral Plaque Microbiome Signatures for Dental Implant Diseases Identified by Strain-Resolution Metagenomics. Npj Biofilms Microbiomes.

[41] Aleksandrowicz P., Brzezińska-Błaszczyk E., Dudko A., Agier J. (2020). Archaea Occurrence in the Subgingival Biofilm in Patients with Peri-Implantitis and Periodontitis. Int. J. Periodontics Restor. Dent..

[42] Yu X., Chan Y., Zhuang L., Lai H., Lang N.P., Keung Leung W., Watt R.M. (2019). Intra-oral Single-site Comparisons of Periodontal and Peri-implant Microbiota in Health and Disease. Clin. Oral Implant. Res..

[43] Kröger A., Hülsmann C., Fickl S., Spinell T., Hüttig F., Kaufmann F., Heimbach A., Hoffmann P., Enkling N., Renvert S. (2018). The Severity of Human Peri-implantitis Lesions Correlates with the Level of Submucosal Microbial Dysbiosis. J. Clin. Periodontol..

[44] Gao X., Zhou J., Sun X., Li X., Zhou Y. (2018). Diversity Analysis of Subgingival Microbial Bacteria in Peri-Implantitis in Uygur Population. Medicine.

[45] Daubert D., Pozhitkov A., McLean J., Kotsakis G. (2018). Titanium as a Modifier of the Peri-Implant Microbiome Structure. Clin. Implant. Dent. Relat. Res..

[46] Al-Ahmad A., Muzafferiy F., Anderson A.C., Wölber J.P., Ratka-Krüger P., Fretwurst T., Nelson K., Vach K., Hellwig E. (2018). Shift of Microbial Composition of Peri-Implantitis-Associated Oral Biofilm as Revealed by 16S rRNA Gene Cloning. J. Med. Microbiol..

[47] Sousa V., Nibali L., Spratt D., Dopico J., Mardas N., Petrie A., Donos N. (2017). Peri-Implant and Periodontal Microbiome Diversity in Aggressive Periodontitis Patients: A Pilot Study. Clin. Oral Implant. Res..

[48] Yu X.-L., Chan Y., Zhuang L.-F., Lai H.-C., Lang N.P., Lacap-Bugler D.C., Leung W.K., Watt R.M. (2016). Distributions of Synergistetes in Clinically-Healthy and Diseased Periodontal and Peri-Implant Niches. Microb. Pathog..

[49] Shiba T., Watanabe T., Kachi H., Koyanagi T., Maruyama N., Murase K., Takeuchi Y., Maruyama F., Izumi Y., Nakagawa I. (2016). Distinct Interacting Core Taxa in Co-Occurrence Networks Enable Discrimination of Polymicrobial Oral Diseases with Similar Symptoms. Sci. Rep..

[50] Tsigarida A.A., Dabdoub S.M., Nagaraja H.N., Kumar P.S. (2015). The Influence of Smoking on the Peri-Implant Microbiome. J. Dent. Res..

[51] Jakobi M., Stumpp S., Stiesch M., Eberhard J., Heuer W. (2015). The Peri-Implant and Periodontal Microbiota in Patients with and without Clinical Signs of Inflammation. Dent. J..

[52] Zheng H., Xu L., Wang Z., Li L., Zhang J., Zhang Q., Chen T., Lin J., Chen F. (2015). Subgingival Microbiome in Patients with Healthy and Ailing Dental Implants. Sci. Rep..

[53] Schaumann S., Staufenbiel I., Scherer R., Schilhabel M., Winkel A., Stumpp S.N., Eberhard J., Stiesch M. (2014). Pyrosequencing of Supra- and Subgingival Biofilms from Inflamed Peri-Implant and Periodontal Sites. BMC Oral Health.

[54] Maruyama N., Maruyama F., Takeuchi Y., Aikawa C., Izumi Y., Nakagawa I. (2014). Intraindividual Variation in Core Microbiota in Peri-Implantitis and Periodontitis. Sci. Rep..

[55] Tamura N., Ochi M., Miyakawa H., Nakazawa F. (2013). Analysis of Bacterial Flora Associated with Peri-Implantitis Using Obligate Anaerobic Culture Technique and 16S rDNA Gene Sequence. Int. J. Oral Maxillofac. Implant..

[56] da Silva E.S.C., Feres M., Figueiredo L.C., Shibli J.A., Ramiro F.S., Faveri M. (2014). Microbiological Diversity of Peri-Implantitis Biofilm by Sanger Sequencing. Clin. Oral Implant. Res..

[57] Koyanagi T., Sakamoto M., Takeuchi Y., Ohkuma M., Izumi Y. (2010). Analysis of Microbiota Associated with Peri-Implantitis Using 16S rRNA Gene Clone Library. J. Oral Microbiol..

[58] Faveri M., Gonçalves L.F.H., Feres M., Figueiredo L.C., Gouveia L.A., Shibli J.A., Mayer M.P.A. (2011). Prevalence and Microbiological Diversity of Archaea in Peri-Implantitis Subjects by 16S Ribosomal RNA Clonal Analysis: Archaea in Peri-Implantitis Subjects. J. Periodontal Res..

[59] Valiente-Mullor C., Beamud B., Ansari I., Francés-Cuesta C., García-González N., Mejía L., Ruiz-Hueso P., González-Candelas F. (2021). One Is Not Enough: On the Effects of Reference Genome for the Mapping and Subsequent Analyses of Short-Reads. PLoS Comput. Biol..

[60] Nie J., Zhang Q., Zheng H., Xu L., Wang X., Chen F. (2020). Pyrosequencing of the Subgingival Microbiome in Peri-implantitis after Non-surgical Mechanical Debridement Therapy. J. Periodontal Res..

[61] Goodrich J.K., Di Rienzi S.C., Poole A.C., Koren O., Walters W.A., Caporaso J.G., Knight R., Ley R.E. (2014). Conducting a Microbiome Study. Cell.

[62] Li J., Quinque D., Horz H.-P., Li M., Rzhetskaya M., Raff J.A., Hayes M.G., Stoneking M. (2014). Comparative Analysis of the Human Saliva Microbiome from Different Climate Zones: Alaska, Germany, and Africa. BMC Microbiol..

[63] Nasidze I., Li J., Schroeder R., Creasey J.L., Li M., Stoneking M. (2011). High Diversity of the Saliva Microbiome in Batwa Pygmies. PLoS ONE.

[64] Mason M.R., Nagaraja H.N., Camerlengo T., Joshi V., Kumar P.S. (2013). Deep Sequencing Identifies Ethnicity-Specific Bacterial Signatures in the Oral Microbiome. PLoS ONE.

[65] Wade W.G. (2013). The Oral Microbiome in Health and Disease. Pharmacol. Res..

[66] Cruz G.D., Chen Y., Salazar C.R., Le Geros R.Z. (2009). The Association of Immigration and Acculturation Attributes with Oral Health among Immigrants in New York City. Am. J. Public Health.

[67] Menon R.K., Gopinath D. (2018). Eliminating Bias and Accelerating the Clinical Translation of Oral Microbiome Research in Oral Oncology. Oral Oncol..

[68] Berglundh T., Armitage G., Araujo M.G., Avila-Ortiz G., Blanco J., Camargo P.M., Chen S., Cochran D., Derks J., Figuero E. (2018). Peri-Implant Diseases and Conditions: Consensus Report of Workgroup 4 of the 2017 World Workshop on the Classification of Periodontal and Peri-Implant Diseases and Conditions. J. Clin. Periodontol..

[69] Khammissa R.A.G., Feller L., Meyerov R., Lemmer J. (2012). Peri-Implant Mucositis and Peri-Implantitis: Clinical and Histopathological Characteristics and Treatment. SADJ.

[70] Salvi G.E., Cosgarea R., Sculean A. (2017). Prevalence and Mechanisms of Peri-Implant Diseases. J. Dent. Res..

[71] Sahrmann P., Gilli F., Wiedemeier D.B., Attin T., Schmidlin P.R., Karygianni L. (2020). The Microbiome of Peri-Implantitis: A Systematic Review and Meta-Analysis. Microorganisms.

[72] Carvalho É.B.S., Romandini M., Sadilina S., Sant’Ana A.C.P., Sanz M. (2023). Microbiota Associated with Peri-Implantitis-A Systematic Review with Meta-Analyses. Clin. Oral Implant. Res..

[73] Jeon Y.D., Lim Y.S., Lee S., Kim K.M., Ryu C.-M., Jung I.Y., Ahn M.-Y., Ann H.W., Ahn J.Y., Ku N.S. (2015). A Comparison Between Next-Generation Sequencing and Bacterial Culture for the Detection of Bacteria in Clinical Specimen. Open Forum Infect. Dis..

[74] Belibasakis G.N., Manoil D. (2021). Microbial Community-Driven Etiopathogenesis of Peri-Implantitis. J. Dent. Res..

[75] Hashimoto Y., Okada S., Yasuda K., Kawagoe M., Kajiya M., Tsuga K. (2022). Microbial Differences between Active and Remission Peri-Implantitis. Sci. Rep..

[76] Hutton B., Salanti G., Caldwell D.M., Chaimani A., Schmid C.H., Cameron C., Ioannidis J.P.A., Straus S., Thorlund K., Jansen J.P. (2015). The PRISMA Extension Statement for Reporting of Systematic Reviews Incorporating Network Meta-Analyses of Health Care Interventions: Checklist and Explanations. Ann. Intern. Med..

[77] Page M.J., McKenzie J.E., Bossuyt P.M., Boutron I., Hoffmann T.C., Mulrow C.D., Shamseer L., Tetzlaff J.M., Akl E.A., Brennan S.E. (2021). The PRISMA 2020 Statement: An Updated Guideline for Reporting Systematic Reviews. Syst. Rev..

[78] ROBINS-E Development Group (2023). Risk Of Bias In Non-randomized Studies-of Exposure (ROBINS-E). Launch Version.

